# Chloroquine alleviates the heat-induced to injure via autophagy and apoptosis mechanisms in skin cell and mouse models

**DOI:** 10.1371/journal.pone.0272797

**Published:** 2022-08-31

**Authors:** Sheau-Chung Tang, Jiunn-Liang Ko, Chun-Te Lu, Pui-Ying Leong, Chu-Chyn Ou, Chih-Ting Hsu, Yu-Ping Hsiao

**Affiliations:** 1 Department of Nursing, National Taichung University of Science and Technology, Taichung, Taiwan; 2 Department of Medical Oncology and Chest Medicine, Chung Shan Medical University Hospital, Taichung, Taiwan; 3 Institute of Medicine, School of Medicine, Chung Shan Medical University, Taichung, Taiwan; 4 Division of Plastic and Reconstructive Surgery, Department of Surgery, Taichung Veterans General Hospital, Taichung, Taiwan; 5 Institute of Medicine, School of Medicine, College of Medicine, National Yang Ming Chiao Tung University, Taipei, Taiwan; 6 Department of Rheumatology, Chung Shan Medical University Hospital, Taichung, Taiwan; 7 Department of Nutrition, Chung Shan Medical University, Taichung, Taiwan; 8 Department of Nutrition, Chung Shan Medical University Hospital, Taichung, Taiwan; 9 Department of Dermatology, Chung Shan Medical University Hospital, Taichung, Taiwan; New York University Langone Health, UNITED STATES

## Abstract

Burns can cause cell death and irreversible tissue damage. We examined the pathway of human dermis fibroblasts cell death caused by skin burns and the roles of chloroquine in human skin keratinocytes HaCaT wound healing. Western blot assays were performed to assess expression of proteins associated with autophagy, apoptosis, and endoplasmic reticulum stress in skin cells following burns. Changes in apoptosis-related proteins were assessed using flow cytometry, and wound cell migration was examined using wound healing assays. The burn animal model was used to test whether chloroquine would promote wound healing. In human burned fibroblasts, expression of LC3B-II and Cleave-caspase-7 was increased, whereas expression of Beclin-1, p62, and Grp78 was decreased. Severe burn induced ER stress and ERK phosphorylation, but PD98059 or necrostatin-1 treatment cells did not affect expression of autophagy LC3B-II protein and can induce apoptosis. Even though added with TGF-β and FGF did not repair autophagy caused by burns. Suggesting that autophagy and apoptosis were involved in heat-injured mechanism. Recombinant Wnt3a protein can help restore expression of β-catenin which reduced following burns in keratinocytes. Wnt3a protein can promote migration of keratinocytes after burns. Interesting, chloroquine increased expression of LC3B-II protein and restored cell migration activity after 24 h of burns. Consistently, surgical dressing containing chloroquine promoted wound healing in a burn animal mode. Autophagy and Wnt/β-catenin is two signalling pathways that participate in cell repair and wound healing in human fibroblasts, keratinocytes. Surgical dressing containing chloroquine can recover wound healing in burned rats.

## Introduction

Burns can cause serious injuries and death, and the severity of burns depends on the affected area and on injury depth [1]. Burns may affect muscles, bones, blood vessels, dermis, and epidermis, which can cause nerve dysfunction resulting in loss of the sense of touch or pain and in serious complications including shock, infection, electrolyte imbalance, or respiratory failure [2]. Burns are graded based on the depth of skin injury [3]. Most commonly, burns in adults are assessed by calculating wound surface area using the Wallace rule of nines [4]. Seeking natural and safe medicines or materials for burns and scalds is an important issue. We therefore aimed to elucidate damage mechanisms in burned and skin and to identify potential treatment drugs.

On cellular level, environmental stress, DNA damage, burns, and will cause endoplasmatic reticulum (ER) stress, which will lead to abnormal cellular calcium secretion and DNA damage, eventually causing apoptosis [5]. Autophagy, however, is a mode of cellular self-protection so as to improve fitness by removing abnormally folded proteins and damaged organelles [6]. When lysosomes are produced during autophagy, autolysosomes are formed which are then surrounded by damaged and decomposed cells. Some studies suggested that autophagy may be a protective mechanism to avert apoptosis [7, 8]; however, further research is required to explore the burns and processes of apoptosis and autophagy.

Previous studies on animal models confirmed that 6 h after burns were induced, expression of Bax, an apoptotic index protein in skin tissue, gradually increases, whereas expression of the Bel-2 protein gradually decreases, indicating that apoptosis occurs in skin cells after 6 h [9]. Other studies found that the ratio of LC3BI/II and expression of Beclin-1, which are autophagy index proteins in burned animal tissue, initially decrease and then gradually recover after 24 h; moreover, TUNEL staining results showed that apoptosis caused by burns initially increases and then decreases over the course of 48 h [10]. Studies confirmed that apoptosis and autophagy are involved in cellular responses of burned tissue [1, 4]. This means that there will be different stages of cellular responses after the burn. The underlying mechanisms, however, remain to be elucidated.

We here explored the following topics to examine the relationship between these mechanisms: first, human skin cells were exposed to high temperatures, and an animal burn model was established to simulate the process of burns and to study healing mechanisms in burn wounds; second, the relationships among cell death, ER stress, and calcium ion in burns was studied by treatments with autophagy inhibitors, necrosis inhibitors, calcium chelators, and growth factors to achieve skin healing. Third, drug compounds for treating burn wounds such as chloroquine, silver sulfadiazine, and 3-methyladenine were compared to determine whether chloroquine would accelerate healing of burned skin and promote skin repair and migration after burns.

## Materials and methods

### Reagents

Dulbecco’s modified Eagle’s medium, foetal bovine serum, and penicillin streptomycin were purchased from Gibco BRL (Grand Island, NY, USA). Chloroquine was purchased from Sigma (St. Louis, MO, USA; C-6628). Anti-phospho-Erk1/2 (#9101), anti-cleaved caspase-7 (#9491), and anti-LC3B (#3868) and were purchased from Cell Signalling (Beverly, MA). Anti-β-actin (A4700) was purchased from Sigma-Aldrich (St. Louis, MO, USA).

### Animal burn model

Total 35 male Wistar of 6- to 8-week postnatal age, purchased from BioLASCO Co (Taiwan) were used in this study which was approved by the Institutional Animal Care and Use Committee of the Chung Shan Medical University Experimental Animal Center, as described previously [11]. Total animals were split 2 group, each group have 5 animals and repeated the independent experiments 3 times. One day before the experiments, hair was removed from the rats’ backs using electric clippers. The back of each animal was marked with India ink, dividing it into six squares. For the burn treatment, the rats were anaesthetized by intraperitoneal injection with pentobarbital (30–60 mg/kg). A steel sheet was heated to 100°C using boiling water and was applied to the rats’ dorsal skin for 15 s to produce a 1-cm^2^ burn wound. Six burn wounds were produced on each rat, as described previously [12–14]. After this treatment, Vaseline, chloroquine, or silver sulfadiazine dressings were applied to the burn wounds three times per day. The rats were housed individually. All animal experimental protocols were approved by ethics committee of Chung-Shan Medical University Experimental Animal Center (Institutional Animal Care and Use Committee, IACUC approval number: 2234).

### Cell cultures and burn injury treatments

Hs68 human foreskin fibroblast cells and HaCaT human keratinocyte cells were acquired from the Food Industry Research and Development Institute, Taiwan. The fibroblasts were used to clarify the mechanism of thermal damage and the keratinocytes were used to observe the wound healing process. Cells were cultured as previously described [11]. In *in vitro* heat shock assay, culture flasks were stoppered and sealed using parafilm and were then placed in a water bath at 50°C for 2, 5, or 10 min, as previously described [15]. After this treatment, the flasks were returned to the incubator for 10 min and were then treated with or without chemicals for 0, 24, or 48 h at 37°C.

### Wound healing assay

Silicon inserts (ibidi GmbH, Gräfeling, Germany) were used for cell seeding in two individual wells in the wound closure seeding model. After 24 h, the culture insert was removed, and the wound was heated in a water bath at 50°C for 5 min. After this treatment, the plates were returned to the incubator for 10 min and were then treated with or without chemicals for 0, 24, or 48 h at 37°C.

### Western blot assay

Cells were extracted with RIPA buffer (Millipore) to yield cell lysates. Anti-phospho-ERK1/2, anti-Grp78, anti-cleaved caspase-7, anti-p62, anti-LC3B, anti-β-catenin, and anti-β-actin were used to detect expressions of phospho-ERK1/2 (42, 44 kDa), anti-Grp78 (also termed heat-shock protein [HSP]-70) (72 kDa), cleaved caspase-7 (18 kDa), anti-p62 (53 kDa), LC3B-I (14 kDa), LC3B-II (17 kDa), β-catenin (80 kDa), and β-actin (42 kDa), respectively. The complete protocol of the western blot analysis and of the annexin V-FITC apoptosis assay were published previously [11]. HRP-conjugated rabbit anti-goat IgG antibody (GeneTex), HRP-linked anti-mouse, and anti-rabbit IgG antibody (Cell Signaling) were used as secondary antibodies. “Intensity of gray scale image was analysed using Image J software. Under the same conditions, the expression level of the protein was corrected to its own β-actin. Then, we compared the obtained value with the control group and showed the value as a bar graph.”

### Statistical analyses

Results are reported as means ± standard deviation. Statistical analyses were carried out using Student’s *t*-tests. Statistical significance is reported at *p* < 0.05. All experiments were performed in triplicates.

## Results

### Burns induce ER stress in human fibroblasts and expression of autophagy-related protein

To evaluate the role of heat shock protein in the burn model, we checked the expression of the ER stress index protein Grp78. After heat-injury and culturing for 24 h, we observed that Grp78 protein expression level increased by 1.95 (heated for 2 min) compared to the control group. However, heat injury for 10 mines caused cell death and degraded the protein (decreased to 0.25 holds compared to the control group).

**Fig 1:**
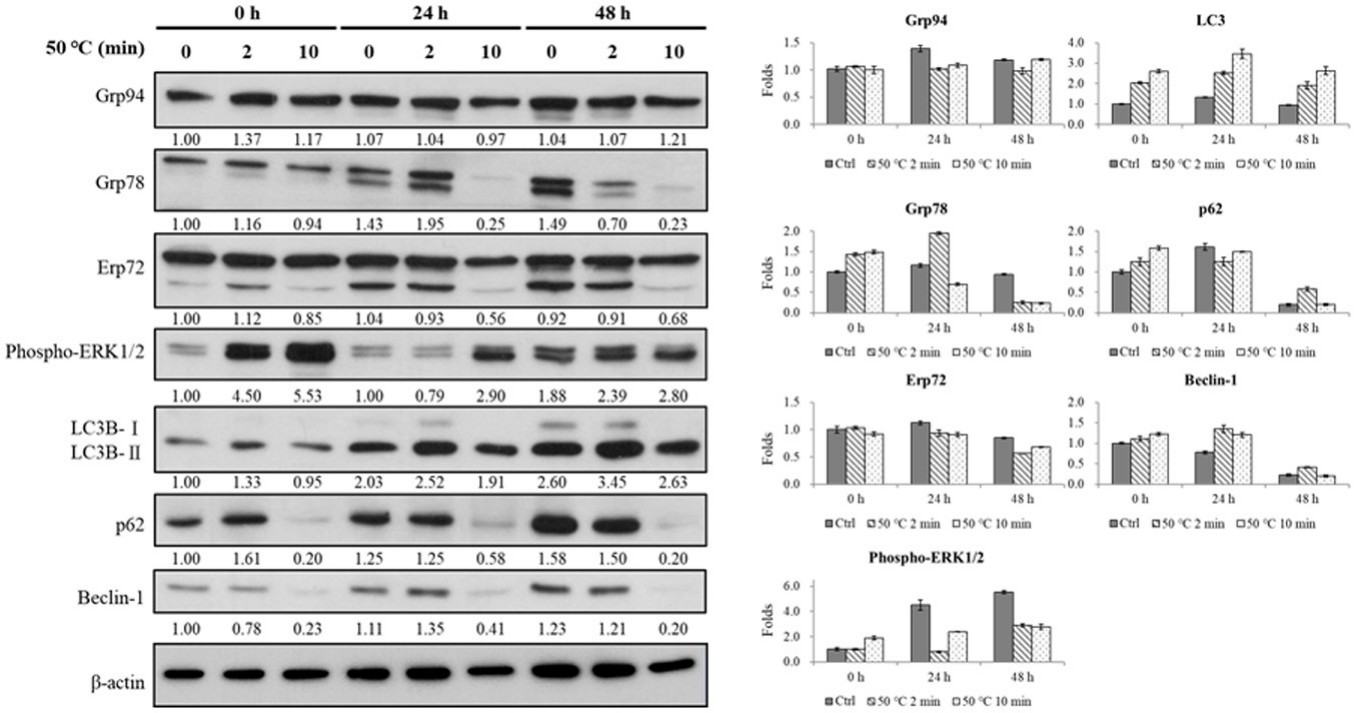
Protein expression responses to heat-induced stress. Human fibroblast Hs68 cells (5 × 10^5^ cells/25T flask) heated at 50°C in a water bath (continued 2 or 10 mins) and keep cultured for 24, or 48 h (0 h: heated and immediately harvested cell). Differences between treatments and controls were considered statistically significant at *p* < 0.05. Compared with control group * *p <* 0.05; ** *p <* 0.01; # compared with heat for 2 min group.

The phosphorylation reaction was significantly correlated with time (at 2 and 10 min, the reaction increased by 4.50- and 5.53-fold, respectively, *p* < 0.05; Fig 1). Expression of proteins p62 and Beclin-1 was decreased (p62 from 1.61- to 0.20-fold, *p* < 0.05; Beclin-1 from 0.78- to 0.23-fold). Interesting, expression of autophagy index protein LC3B-II increased significantly after 24 h post-burns time (from 2.52- to 1.91-fold, and from 3.45- to 2.63-fold at 24 and 48 h, respectively).

The medium was replaced immediately after heat treatments at 50°C (5 min) to test whether damaged cells can be repaired under optimal conditions as growth factors in the medium may be damaged by high temperatures. Expression of the above-mentioned index proteins did not change. Those results showed that ERK phosphorylation caused by burns was induced immediately. Burns can induce human fibroblast apoptosis, autophagy, and the expression of endoplasmic reticulum stress-related proteins. With the severity of burns and scalds, it can be observed that autophagy is accompanied by apoptosis.

### Effects of calcium chelator BAPTA-AM or Grp78 recombinant protein on autophagy and apoptosis in human fibroblasts induced by burns

**Fig 2:**
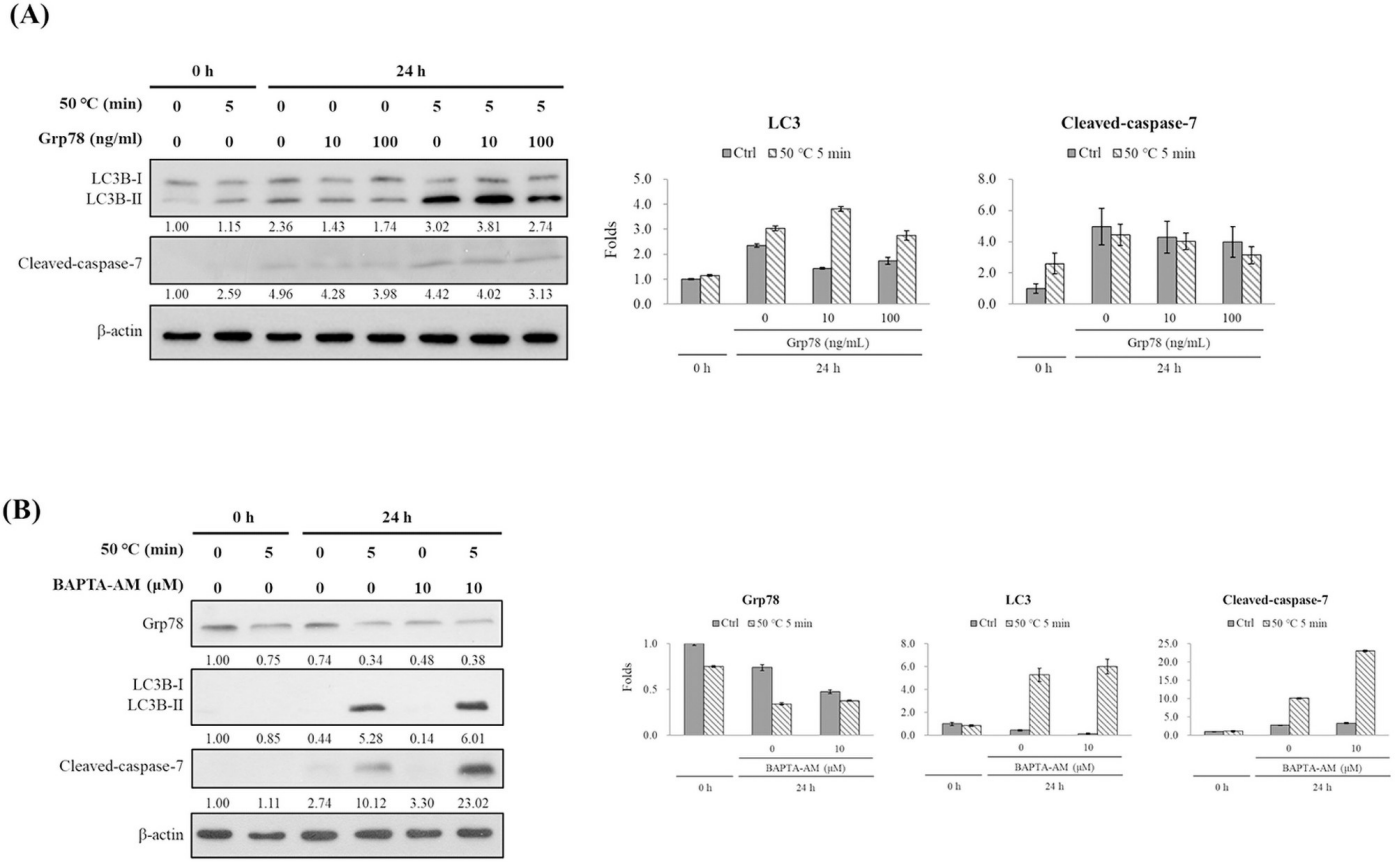
Effect of BAPTA-AM or Grp78 on heat-induced autophagy and apoptosis. Hs68 cells (1.5 × 10^5^ cells/25T flask) were heated to 50°C in a water bath for 5 min. After the heat treatment, cells were treated with a calcium chelator BAPTA-AM (10 μM) or additionally with Grp78 recombinant protein (10 or 100 ng/mL) for 24 h and were then examined by western blotting. The immune reactions were visualized by an ECL and UVP Biospectrum Imaging System (Upland, CA, USA). The immunoreactive bands were quantified using Image J software and shown a bar graph.

ER stress always follow the calcium release. Calcium chelator BAPTA-AM and Grp78 recombinant protein were used to explore the effects of calcium ions on cell death induced by burns. Grp78 was added to burned cells to test whether Grp78 protein can protect cells from apoptosis. Addition of Grp78 recombinant protein produced no effect on expression of LC3B-II and cleaved-caspase-7 (Fig 2A). These results show that removal of calcium ions by a calcium chelator does not affect expression of Grp78 protein and autophagy in burned cells.

BAPTA-AM caused a significant increase in the expression of apoptotic index protein cleaved-caspase-7 (24 h after the heat treatment, it increased from 1.0- to 10.12-fold and increased to 23.02-fold when the calcium chelator was used to remove calcium ions). However, no effect on expression of Grp78 and LC3B-II was observed (Fig 2B). Moreover, burned cells still underwent apoptosis, and Grp78 recombinant protein did not affect autophagy and apoptosis.

### Effects of MERK inhibitor PD98059 on autophagy and apoptosis in human fibroblasts induced by burns

**Fig 3:**
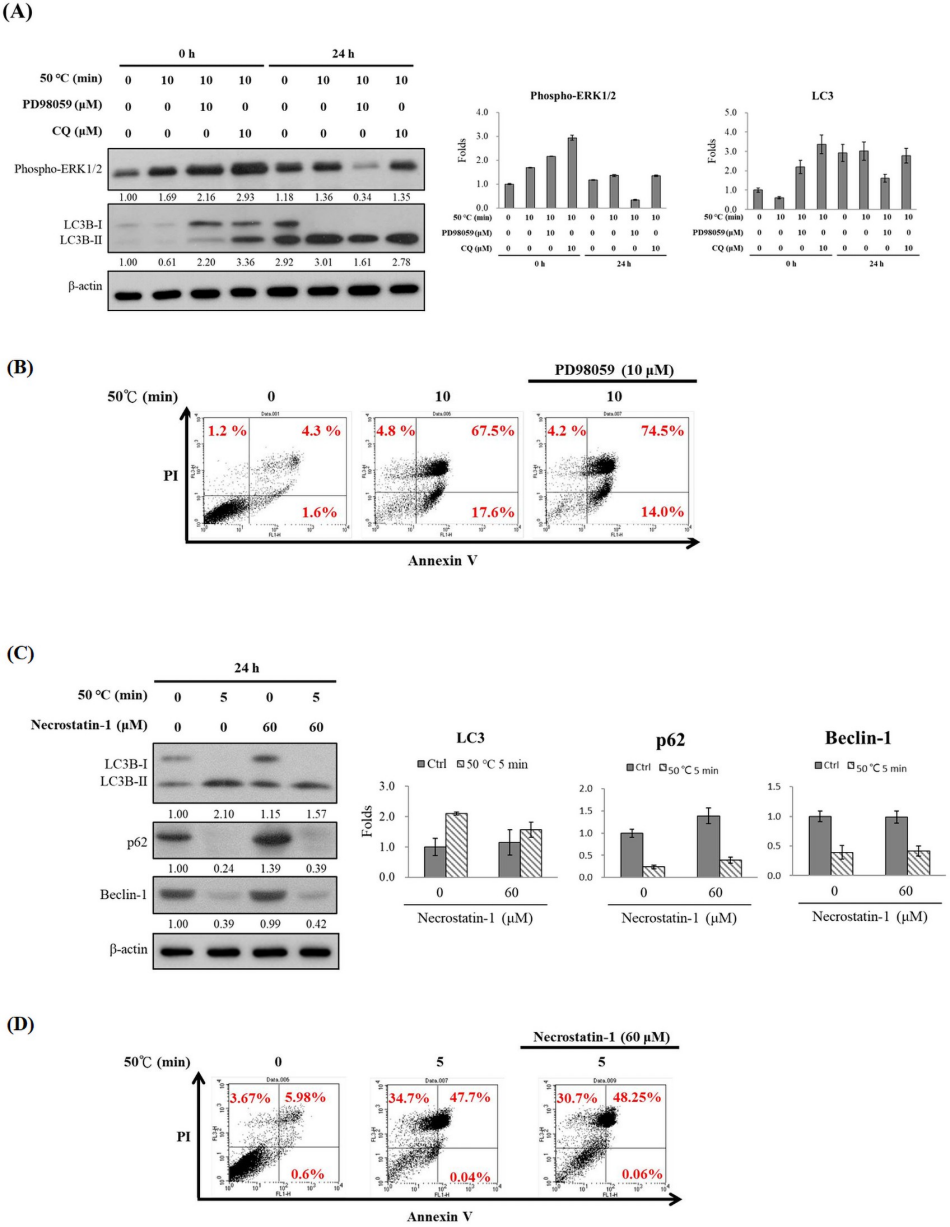
Effect of PD98059 or necrostatin-1 on heat-induced injuries. Hs68 cells (1.5 × 10^5^ cells/25T flask) were heated to 50°C in a water bath (0 or 5 min) and treated with MEK inhibitor PD98059 (0 or 10 μM), with the autophagy inhibitor chloroquine (0 or 10 μM), or with necroptosis inhibitor necrostatin-1 (0 or 60 μM) for 0 or 24 h. Effects of **(A)** PD98059 or **(C)** Necrostatin-1 on protein levels were determined by western blotting. The immunoreactive bands were quantified using Image J software and shown a bar graph. Flow cytometry was used to detect signals of the apoptotic index protein annexin V(+)/PI(+) in the **(B)** PD98059 or **(D)** ecrostatin-1 treatments.

The ERK upstream factor-MERK inhibitor PD98059 was used to explore the effects of ERK phosphorylation on cell death caused by burns and to examine whether apoptosis induced by burns was caused by ERK phosphorylation. A pre-treatment with PD98059 can block ERK phosphorylation (from 1.10- to 0.85-fold, it was decreased by 25%) (Fig 3A), but it exerted no significant impact on expression of LC3B-II. Moreover, PD98059 increased the annexin V(+)/PI(+) signalling induced by burns (67.5% and 74.5%, respectively; Fig 3B), and necrosis due to burns was observed immediately. Our results suggest that blocking cell phosphorylation can induce apoptosis and cannot affect expression of autophagy LC3B-II protein and. Burns thus do not cause apoptosis through a single ERK phosphorylation pathway.

### Effects of necrosis inhibitor necrostatin-1 on autophagy and apoptosis in human fibroblasts induced by burns

The necrosis inhibitor necrostatin-1 was used to test whether it can decelerate cell death caused by necrosis and to explore whether cell death by necrosis is caused by burns. Compared with the burn group, necrostatin-1 showed no significant impact on the expression of LC3B-II (from 2.10- to 1.57-fold), p62 (from 0.24- to 0.39-fold), and Beclin-1 (from 0.39- to 0.42-fold) (Fig 3C). The annexin V(+)/PI(+) signal was simultaneously detected by flow cytometry. Necrostatin-1 reduced the necrosis rate from 34.7% to 30.7% but did not decelerate annexin V(+)/PI(+) signalling caused by burns (Fig 3D). Those results showed that the use of necrostatin-1 blocked the necrosis pathway but did not affect autophagy caused by burns and did not decelerate apoptosis.

### Effects of transforming growth factor (TGF-β), fibroblast growth factor (FGF), and epithelial growth factor (EGF) on autophagy, fibronectin expression, and wound healing following burns

**Fig 4:**
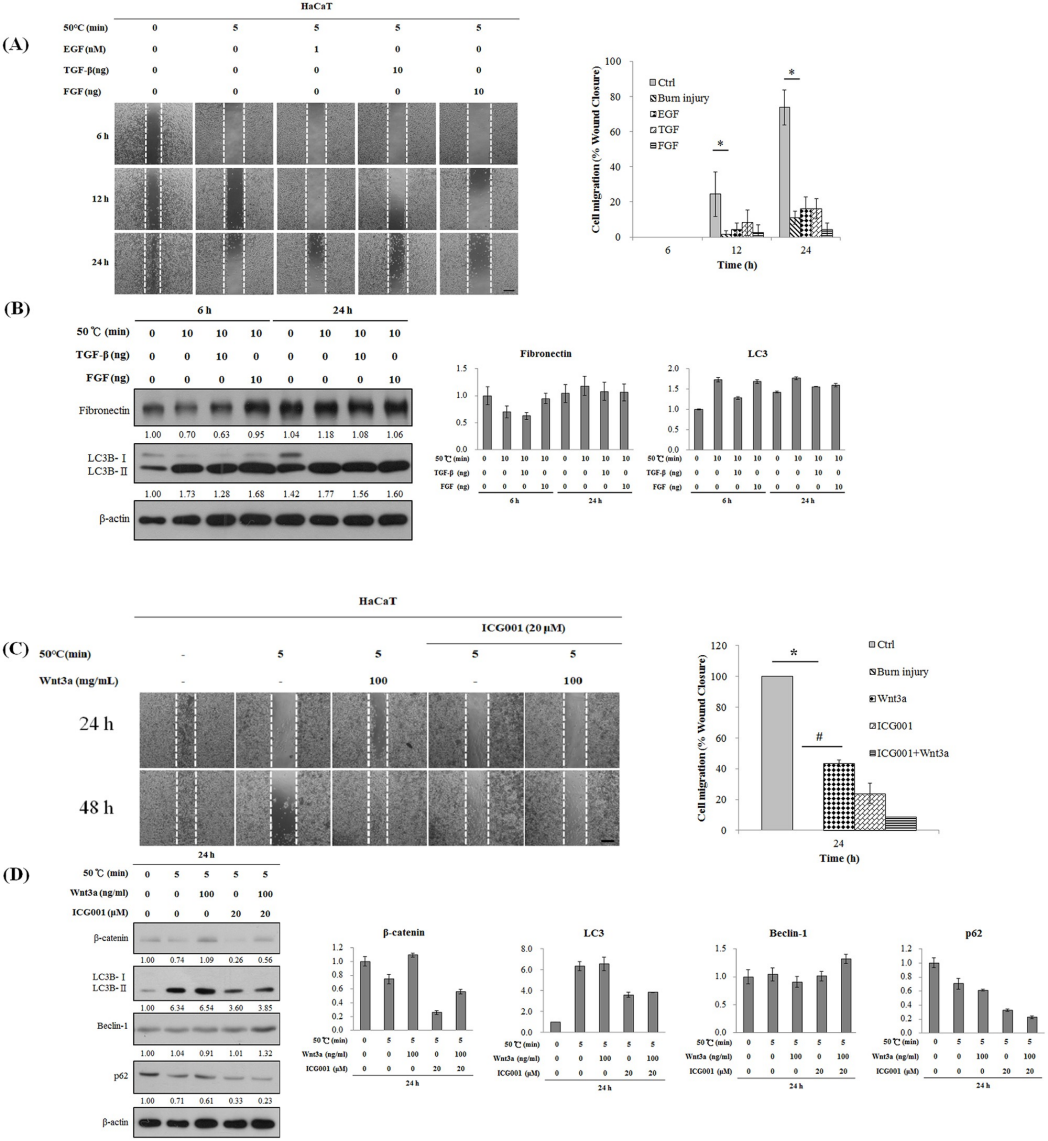
Effect of transforming growth factor-beta or fibroblast growth factor on heat-induced autophagy and migration in skin cells. The Wnt/β-catenin pathway is involved in heat-mediated wound recovery in keratinocytes. A wound healing assay was performed using culture inserts. The rate of wound closure was observed at the indicated times. Gap width of the wounds was measured and recorded and was then compared to the initial gap size at 0 h. **(A)** HaCaT cells (2 × 10^4^ cells/insert) were heated to 50°C in a water bath (0 or 5 min) and treated with EGF (1 nM), TGF-β (10 ng), or FGF (10 ng) for 6, 12 or 24 h. **(B)** Hs68 cells (1.5 × 10^5^ cells/25T flask) were heated to 50°C in a water bath (0 or 10 min) and were treated with TGF-β (0 or 10 ng) or FGF (0 or 10 ng) for 6, 24 h. Protein levels were determined by western blotting. **(C)** HaCaT cells were heated to 50°C in a water bath (0 or 5 min) and were treated with Wnt3a (0 or 100 ng/mL) or β-catenin inhibitor-ICG001 (20 μM) for 0 or 24 h. **(D)** β-catenin expression was determined by western blotting. The immunoreactive bands were quantified using Image J software and shown a bar graph. Scale bar = 200.0 μm.

TGF-β and FGF are important growth factors during healing of burn wounds in epidermis. The migration status of keratinocytes can represent the repair ability of scalded skin. We tested whether TGF-β, FGF, and EGF would promote migration of human keratinocytes after burns and thereby promote wound healing. TGF-β, FGF, and EGF did not restore migration activity of cells within 24 h (Fig 4A). TGF-β and FGF did not affect expression of fibronectin at 24 h (Fig 4B). After TGF and FGF had been administered for 24 h, expression of LC3B-II did not increase. Those results showed that TGF-β and FGF did not significantly affect autophagy caused by burns.

### The effect of Wnt/β-catenin pathway in cell migration after burns

Adding Wnt3a recombinant protein can significantly recover the cell decreased migrate ability caused by burns (Fig 4C). Consistently, Wnt3a recombinant protein significantly increased the expression of β-catenin in burns cells (from 0.74- to 1.09-fold, *p* < 0.05) (Fig 4D). This phenomenon can be inhibited by ICG001 (from 0.74- to 0.26-fold, *p* < 0.05). However, expression of autophagy-related proteins including LC3B-II, p62, and Beclin-1 was not affected by Wnt3a recombinant protein compared with burns cells group. Therefore, the use of Wnt3a recombinant protein in keratinocytes after burns may promote cell migration through the recovery of β-catenin expression but does not prevent autophagy caused by burns.

### Effects of chloroquine on autophagy and apoptosis induced by burns in human dermis fibroblasts

**Fig 5:**
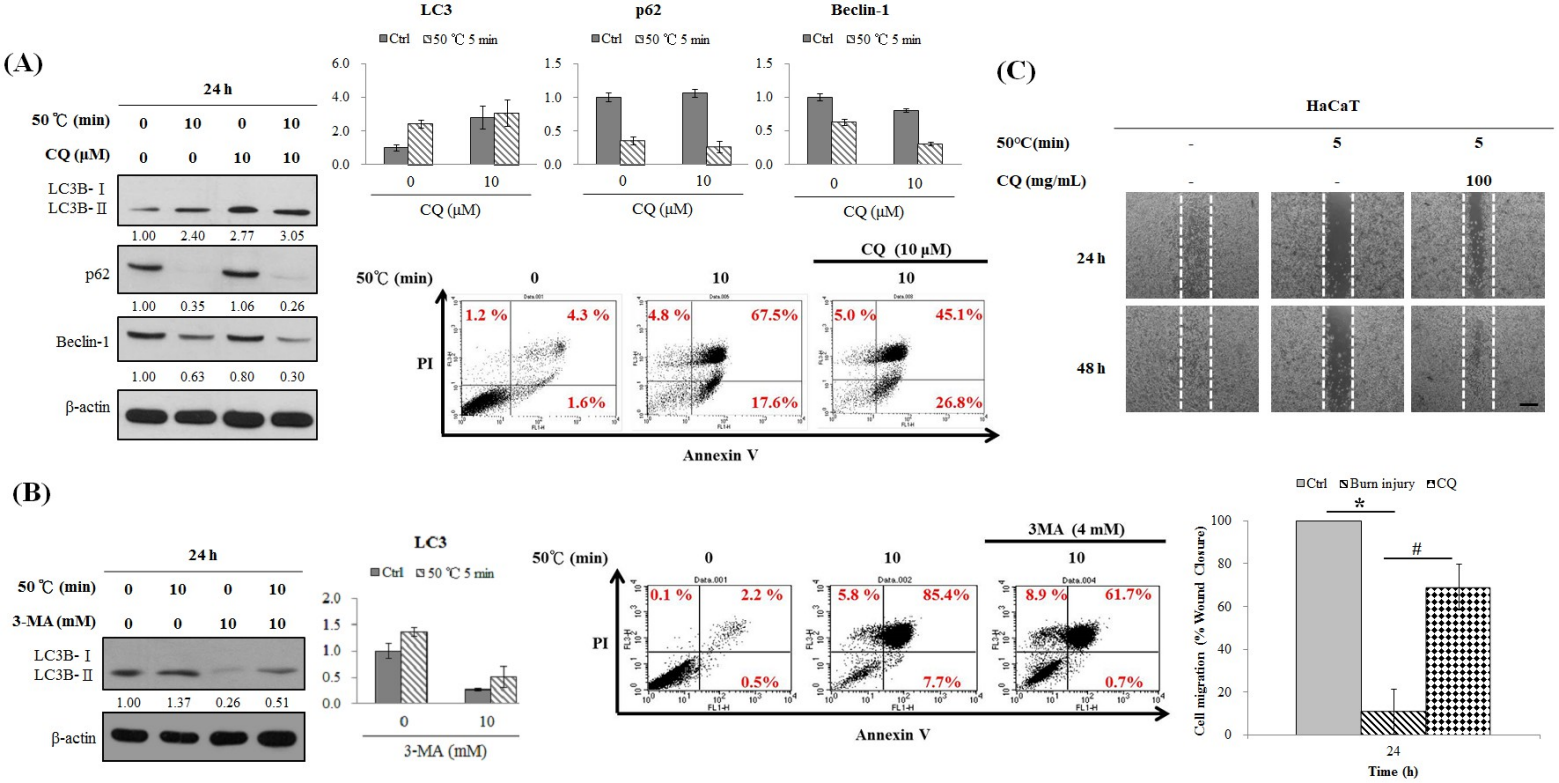
Effect of chloroquine or 3-methyladenine on heat-induced stress and migration in skin cells. Hs68 cells (1.5 × 10^5^ cells/25T flask) were heated to 50°C (0 or 10 min), and autophagy inhibitors chloroquine (10 μM) or 3-methyladenine (4 mM) were added after cooling **(A, B left)**. At 0 and 24 h, western blotting was performed to examine changes in protein expression **(A, B right)**. The immunoreactive bands were quantified using Image J software and shown a bar graph. After 24 h, flow cytometry was used to detect annexin V(+)/PI(+) signals. **(C)** Wound healing assays were performed using culture-inserts. Human keratinocyte HaCaT cells (2 × 10^4^ cells/ insert) were heated to 50°C in a water bath (0 or 5 min) and treated with chloroquine (0 or 10 μM) for 24 and 48 h. Scale bar = 200.0 μm.

Autophagy and apoptosis play opposite roles in the development of burn injury. Chloroquine (CQ) increased expression of LC3B-II protein (from 2.40- to 3.05-fold) and reduced expression of Beclin-1 protein (from 0.63- to 0.30-fold) in burned cells (Fig 5A). 3-Methyladenine (3MA) reduced LC3B-II protein expression (from 1.37- to 0.51-fold) (Fig 5B). Using flow cytometry, CQ and 3MA were observed to decelerate annexin V(+)/PI(+) signalling caused by burns (from 67.5% to 45.1% and from 85.4% to 61.7%, respectively; Fig 5). Therefore, decelerating autophagy caused by burns may also slow down apoptosis-related processes.

To examine the effects of CQ on migration activity of human keratinocytes after burns, and found that CQ restore cell migration activity after 24 h of burns, and 48 h after the burn treatment, the wounds were almost completely closed (Fig 5C).

### The effect of chloroquine on wound healing of burned animal models

**Fig 6:**
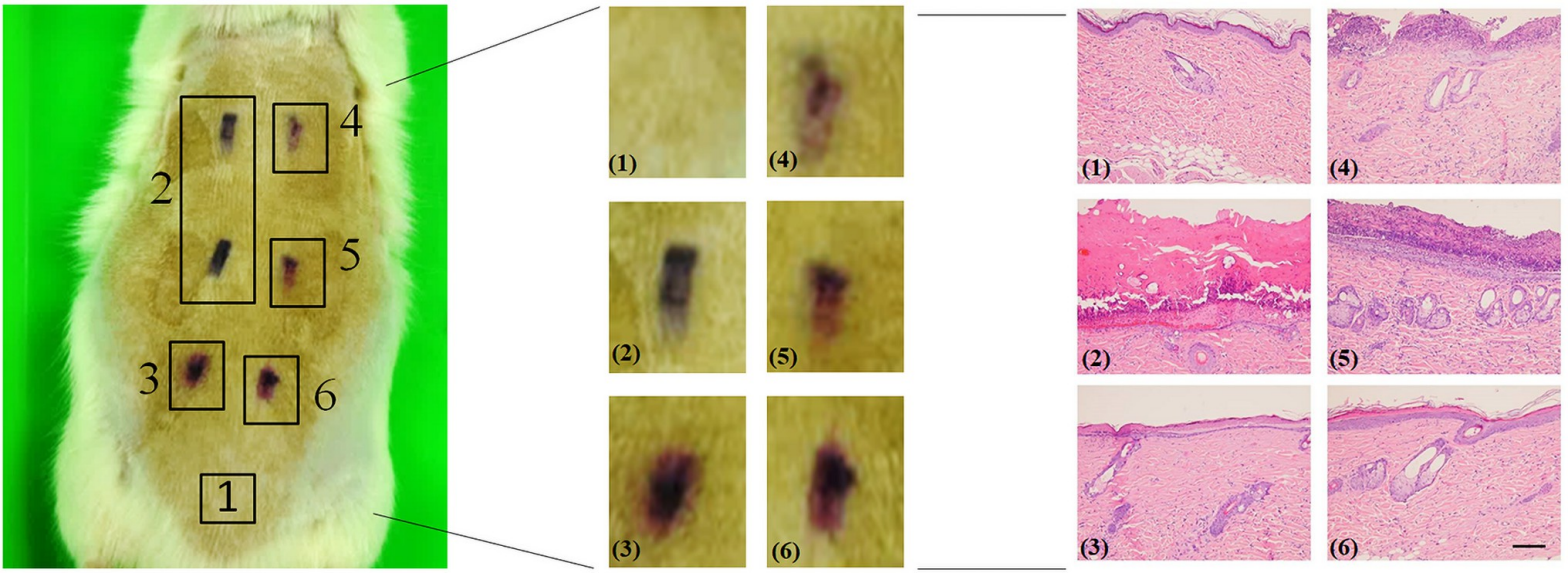
HE stains of rat skin burn. Wistar rats were used for burning and scald experiments, and we investigated whether chloroquine could promote wound healing in burn and scald rats. A steel cube (1 cm^2^) of 100°C was used to burn six parts of the skin on the back of the rat for 10 seconds. Vaseline dressings containing different proportions of chloroquine were applied three times a day, and the rats were sacrificed 48 hours later. The wound site image of Fig 5 middle (magnified 10x). Analysed skin tissue sections. Normal skin (1), Burned skin (2), Silver sulfadiazine 1% cream (3), Vaseline (4), 1% CQ (5), and 5% CQ (6). The burn wound skin and surrounding skin served as a control group (1 cm^2^) fixed in 10% formalin (BNT, 0002A). Sections approximately 4 μm thick were stained with hematoxylin for 10 minutes, washed, and stained with eosin for 2 minutes. Use Leica Autostainer XL ST5010 automatic dyeing machine for dyeing (magnified 100x) (Scale bar = 200.0 μm). The stained-glass slide was checked with an optical microscope.

To clarify whether CQ can promote wound healing in burned rats, dorsal skin tissue sections were analysed. Compared with the control group, leucocyte infiltration was observed in the cuticle of burned epidermis, and hair follicles in the dermis were deformed and showed shallow second-degree burns and burned tissues. Burned tissue treated with Vaseline did not retard heat injury or persistent inflammation. Interestingly, the burned tissue after treatment with 1% CQ, the wound structure was flat and appeared smoother than that of the wounds treated with Vaseline. After treatment with a high concentration of 5% CQ, the wounds were flat and inflammation was reduced, and the shape of hair follicles was restored (Fig 6). Therefore, CQ can decelerate inflammation in burn wound and can promote repair and healing of burn wounds.

## Discussion

We aimed to explore mechanisms of ER stress, autophagy, apoptosis, and necrosis of cells after burns, and we also investigated effects of inhibitors, growth factors, and of the Wnt/β-catenin signalling pathway on fibroblast repair and keratinocyte migration.

Burns can cause different degrees of ER stress, apoptosis, autophagy, and necrosis. When cells are exposed to high temperatures, heat shock can occur, and HSPs are generated. If HSP expression increases continuously, defence mechanisms will be activated to protect cells from high temperatures or other adverse effects [16]. Simultaneously, ER stress is induced and the abnormal unfolded protein response is activated to increase expression of chaperone proteins, such as the 78 kDa glucose-regulated protein (GRP78), to reduce translation of globulin, and to degrade folding proteins [17, 18]. However, if ER stress cannot be relieved for extended periods of time, it will result in apoptosis [17]. Moreover, when ER stress occurs, it may cause abnormal secretion of calcium ions and loss of the calcium storage capacity [19, 20]. GRP78 may cause the release of cytochrome C from granulocytes and subsequently cause apoptosis [20–22]. However, whether autophagy can inhibit ER stress, reduce apoptosis, and promote cell survival by removing abnormal folding protein fragments remains to be elucidated. In the present study, expression of GRP78 decreased after burn treatments, and this decrease was correlated with treatment time (Fig 1). Using the calcium chelator BAPTA-AM, autophagy cannot be regulated after chelating calcium, but apoptosis can be promoted (Fig 2). Few studies so far focused on ER stress in skin cells of burn wounds. Therefore, the mechanisms of cell death in burn wounds, ER stress, and calcium ions demand further research attention.

During wound healing, β-catenin will increase in the proliferation phase to regulate the proliferation rate, motility, and invasiveness of fibroblasts in the dermis [23]. In addition, proliferation of fibroblasts and scar hyperplasia promoted by TGF-β is associated with the regulation of β-catenin [24]. When cells migrate or divide, the cell–cell adhesion protein can be disconnected to support wound healing. The Wnt3a/β-catenin pathway is important for self-renewal of skin stem cells [25]. In the current study, Wnt3a recombinant protein did not restore apoptosis caused by burns (Fig 4D). Therefore, the Wnt3a/β-catenin pathway does not help repair apoptotic damage caused by burns; however, it still plays an important role. Wnt3a recombinant protein appeared to restore shrunken cell morphology caused by burns as observed by microscopy. Furthermore, the cell healing test showed that Wnt3a recombinant protein can promote cell migration. The above-mentioned processes can be inhibited by the β-catenin inhibitor ICG001 (Fig 4C). Previous research showed that when skin fibroblasts and keratinocytes are co-cultured through simulating skin structure, Wnt signalling in fibroblasts activated by Wnt3a protein can promote proliferation of keratinocytes [26]. Therefore, in addition to the fibroblasts discussed in previous literature, the Wnt3a/β-catenin pathway is also of importance for skin keratinocytes.

TGF-β1, TGF-β2, and TGF-β3 can promote migration of fibroblasts and endothelial cells and cause fibroblasts in the extracellular matrix to accumulate in granulation tissue [27]. In the current study, after FGF, TGF-β, and EGF were added to burned Hs68 and HaCaT cells, cell migration and promotion of cell death caused by burns were expected (Fig 4A and 4C). Based on the western blot assay, after 6 h, FGF promoted expression of fibronatin in fibroblasts, whereas 24 h later, its expression did almost not differ from that in normal cells. The effect of FGF on proliferation of fibroblasts cannot be maintained for extended periods of time (Fig 4B). Furthermore, FGF and TGF-β did not prevent autophagy caused by burns, and FGF, TGF-β, or EGF did not promote migration of keratinocytes. Therefore, the main functions of these growth factors require further examination. EGF can be used in two kinds of cells simultaneously. Furthermore, enzyme-linked immunosorbent assays can be used to explore intracellular oxide concentrations increase and to explore the relation of cell death in burn animal models.

Chloroquine has received considerable attention because of its high efficacy and good tolerance in human patients, chloroquine has been used to treat numerous diseases such as malaria, rheumatoid arthritis, cancer, and lupus erythematosus [28–30]. Chloroquine inhibits late autophagy, and it can prevent lysosomes from hydrolysing compounds in its vicinity by increasing the pH value, resulting in considerable accumulation of autolysosomes which inhibits autophagy [31]. In the current study, The standard treatment of mild to moderate burns (such as antibiotic ointments, dressing in silver, and debridement, however, systemic antibiotics are highly associated with mechanisms of resistance, which compromise the treatment process [32].” Human fibroblasts also showed autophagy and apoptosis after burn treatments. Therefore, chloroquine and 3MA were used to explore whether apoptosis can be impeded through regulating autophagy (Fig 5). Chloroquine can promote cell migration, and chloroquine and Vaseline were mixed at different ratios for wound dressings which were applied to superficial second-degree burn wounds of rats. The mixtures of chloroquine and Vaseline can help restore the shape of hair follicles; however, no antibacterial and antibiotic components were used in the dressing; thus, the tissue sections showed leucocyte infiltration (Fig 6). In the animal model, silver sulfadiazine cream was used in the control group [33, 34].

Burns can cause autophagy and apoptosis in skin cells. If autophagy occurs for extended periods of time, its protective functions will be transformed to promoting cell death. Further research on cell death caused by burns and regulation of growth factors through the co-culture system of fibroblasts and keratinocytes would be necessary to help understand microenvironmental processes in of burn wounds as the graphic abstract.

## Conclusion

The results of the study provide the effective concentration of CQ and clarify the mechanisms of the heat-induced skin injure. Autophagy and Wnt/β-catenin pathways participate in cell repair and wound healing due to burns and scalds. The animal model used in this study may help to improve the treatment of burn wounds to reduce pain, accelerate wound healing, and reduce the possibility of scar formation. CQ has the potential to be a safe and effective material for burn and scald ointment.

## Supporting information

S1 Graphical abstract Burns and scalds can cause different degrees of ER stress, apoptosis, autophagy, and necrosis. In this study, we clarify that the autophagy and Wnt/β-catenin participate in cell repair and wound healing in human fibroblasts, keratinocytes. Chloroquine improves wound healing through autophagy and Wnt/β-catenin mechanisms in burned-skin cell and burned-mouse.
(TIF)

S1 Fig Effect of transforming growth factor-beta or fibroblast growth factor on heat-induced autophagy and migration in Hs68 cells. A wound healing assay was performed using culture inserts. The rate of wound closure was observed at the indicated times. Gap width of the wounds was measured and recorded and was then compared to the initial gap size at 0 h. **(A)** Hs cells (2 × 10^4^ cells/insert) were heated to 50°C in a water bath (0 or 5 min) and treated with EGF (1 nM), TGF-β (10 ng), or FGF (10 ng) for 6, 12 or 24 h. Scale bar = 100.0 μm.
(TIF)

S1 Raw images (ZIP)
