# Supplementary figures and images for: Chloroquine alleviates the heat-induced to injure via autophagy and apoptosis mechanisms in skin cell and mouse models
Source: PLoS One. 2022 Aug 31;17(8):e0272797. doi: 10.1371/journal.pone.0272797 (PMC9432730; doi:10.1371/journal.pone.0272797)

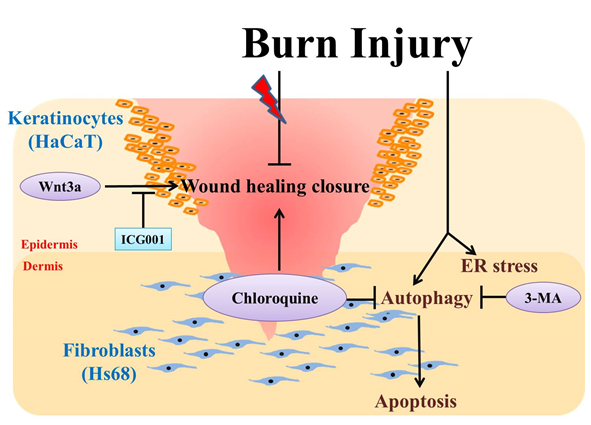

Supplement: S1 Graphical abstract — In this study, we clarify that the autophagy and Wnt/β-catenin participate in cell repair and wound healing in human fibroblasts, keratinocytes. Chloroquine improves wound healing through autophagy and Wnt/β-catenin mechanisms in burned-skin cell and burned-mouse. (TIF) [file pone.0272797.s001.tif]

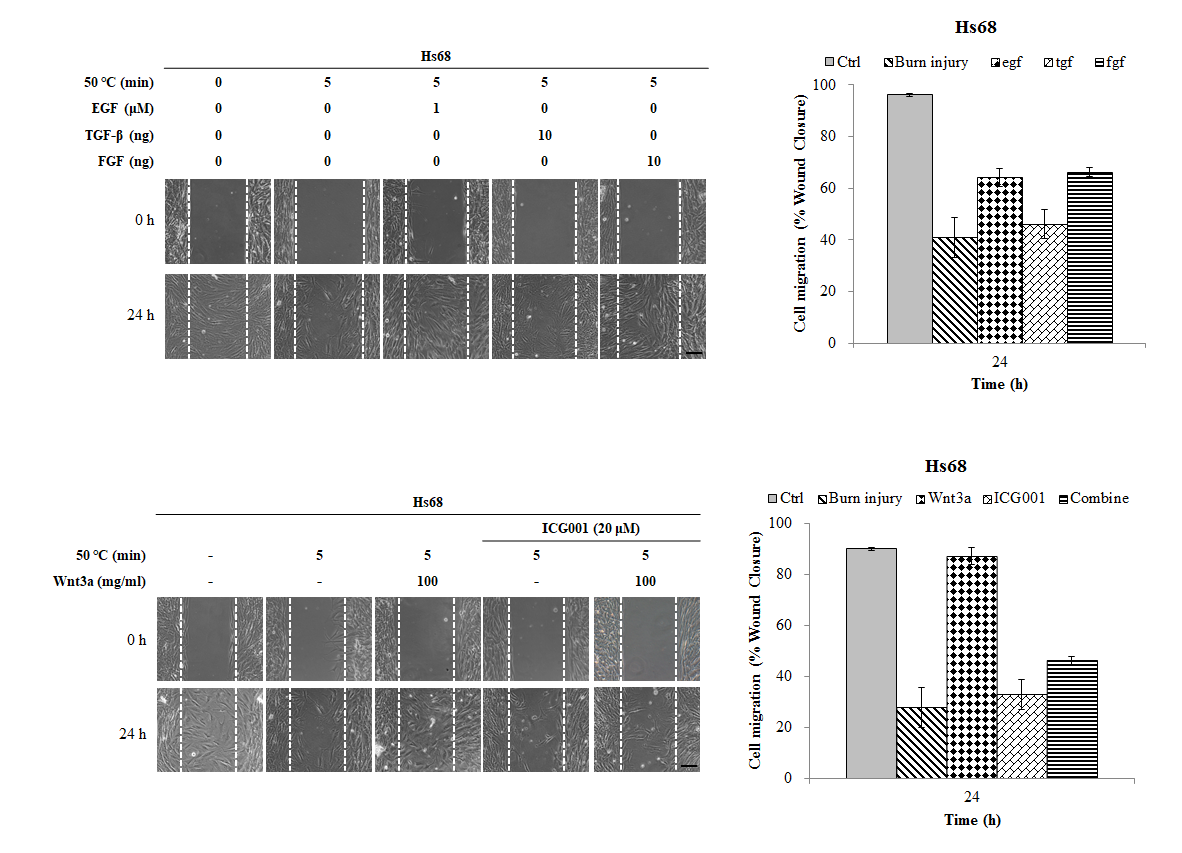

Supplement: S1 Fig — A wound healing assay was performed using culture inserts. The rate of wound closure was observed at the indicated times. Gap width of the wounds was measured and recorded and was then compared to the initial gap size at 0 h. (A) Hs cells (2 × 10^4^ cells/insert) were heated to 50°C in a water bath (0 or 5 min) and treated with EGF (1 nM), TGF-β (10 ng), or FGF (10 ng) for 6, 12 or 24 h. Scale bar = 100.0 μm. (TIF) [file pone.0272797.s002.tif]
