# Supplementary material for: Chloroquine alleviates the heat-induced to injure via autophagy and apoptosis mechanisms in skin cell and mouse models
Source: PLoS One. 2022 Aug 31;17(8):e0272797. doi: 10.1371/journal.pone.0272797 (PMC9432730; doi:10.1371/journal.pone.0272797)

S1_raw_images

Chloroquine alleviates the heat-induced to injure via autophagy and apoptosis mechanisms in skin cell and mouse models

Figure 1.

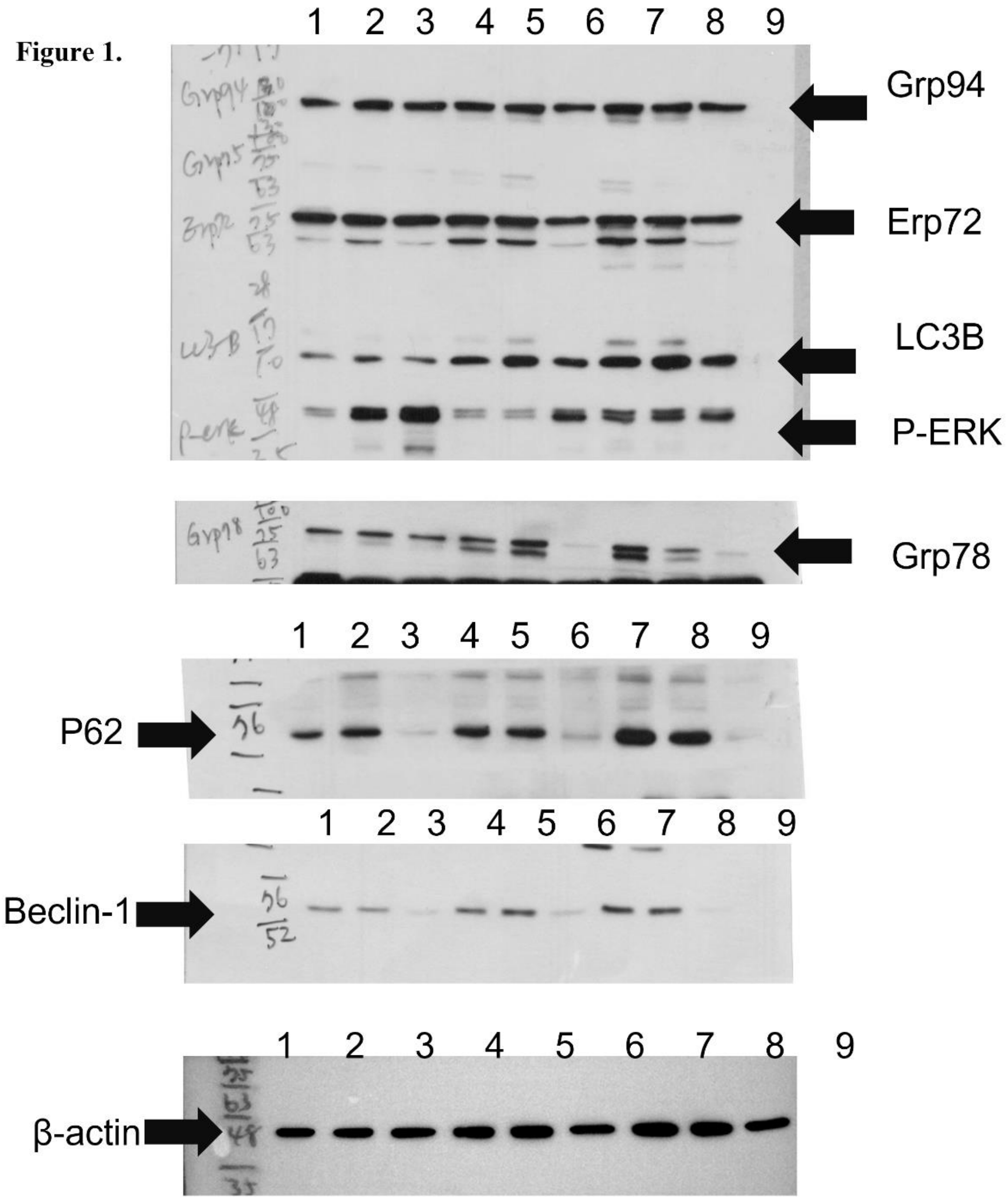

Figure 2. (A)

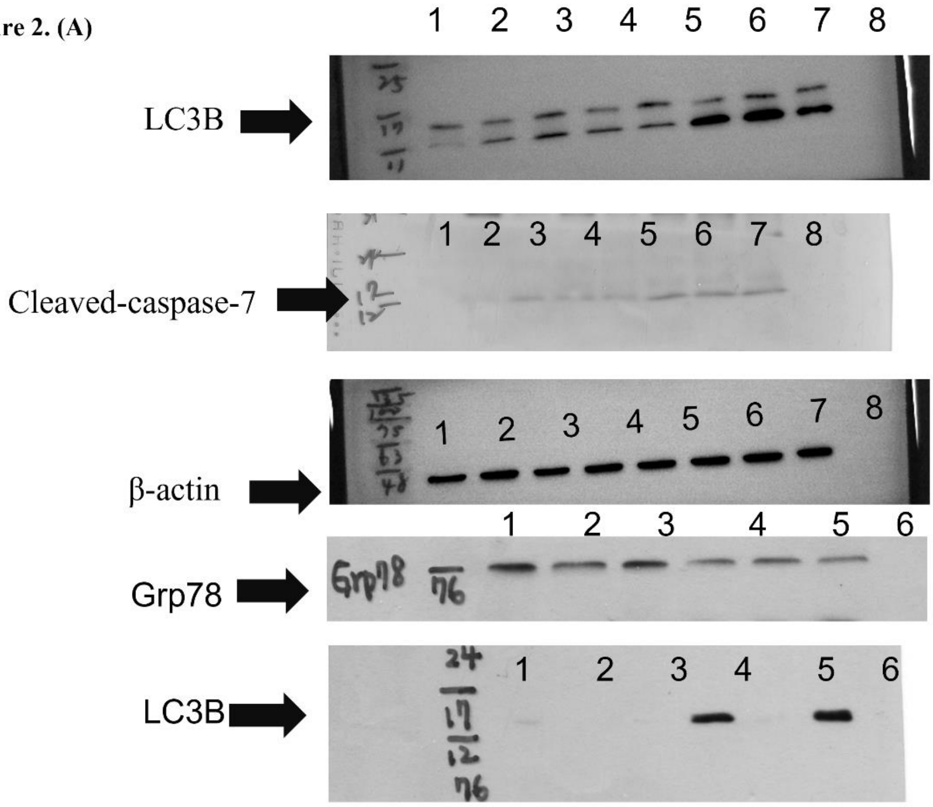

Figure 2. (B)

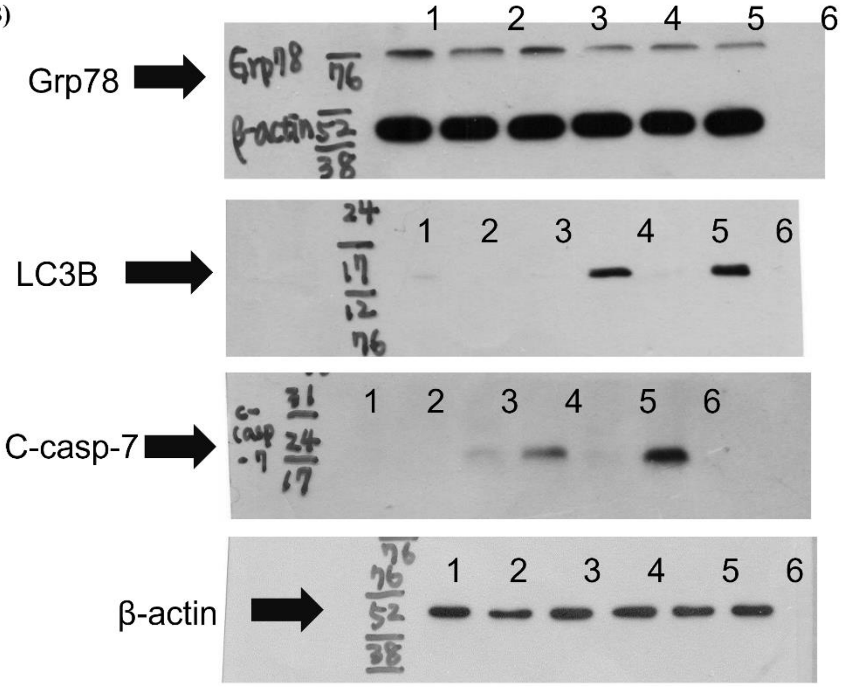

Figure 3. (A)

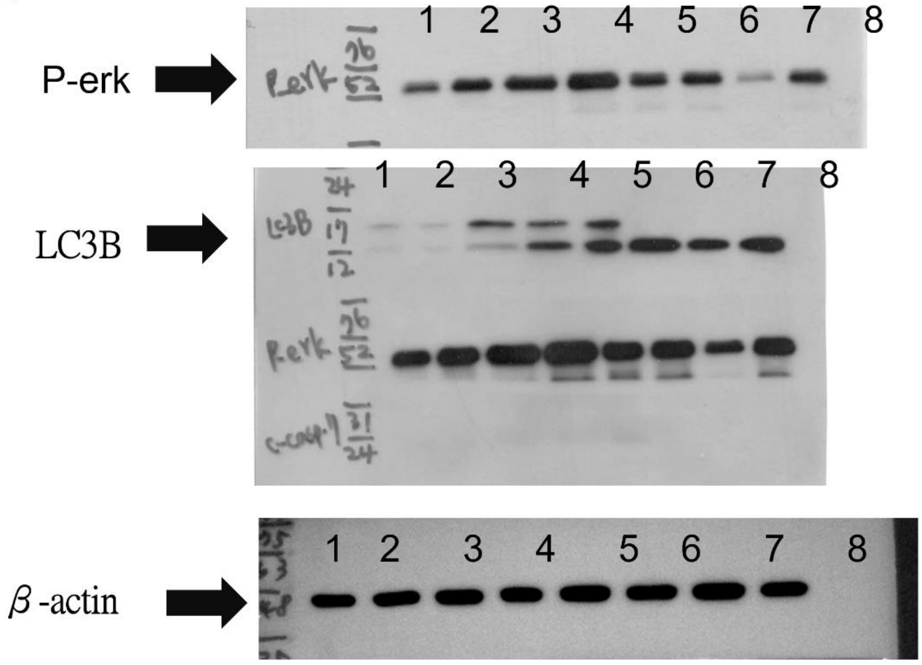

Figure 3. (C)

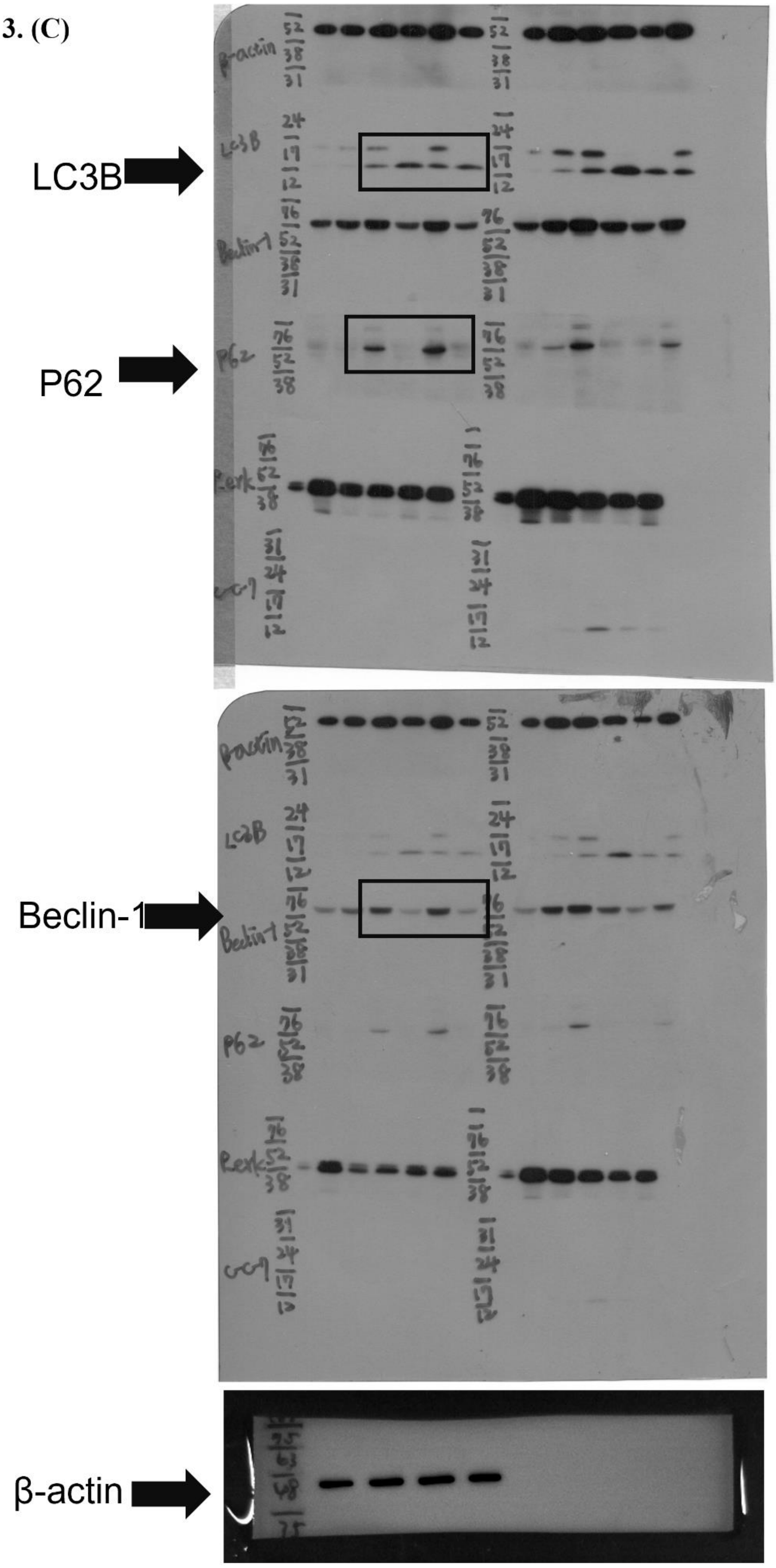

Figure 4 (B).

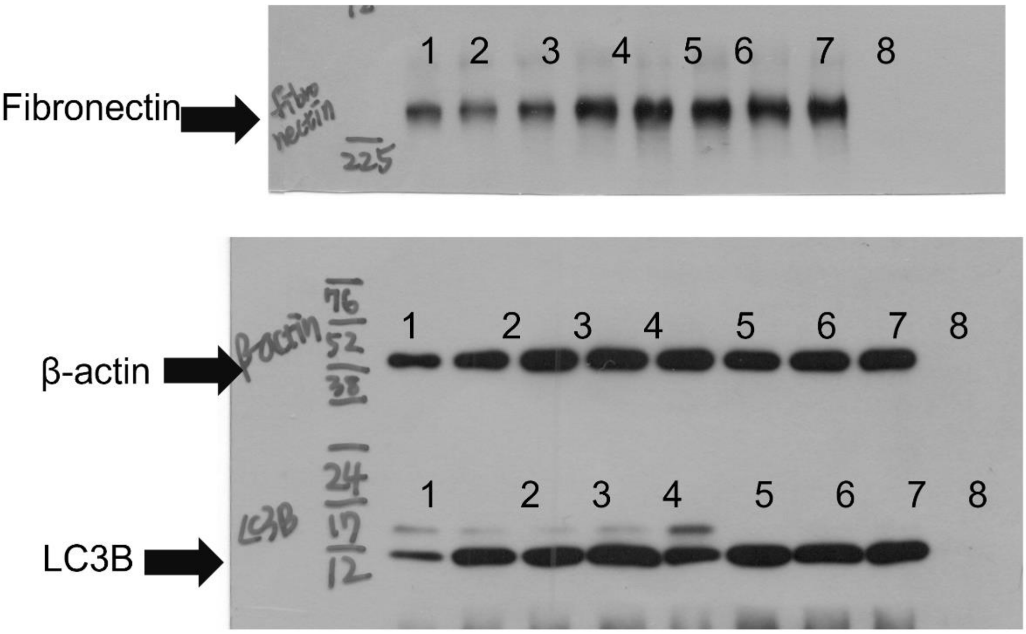

Figure 4 (D).

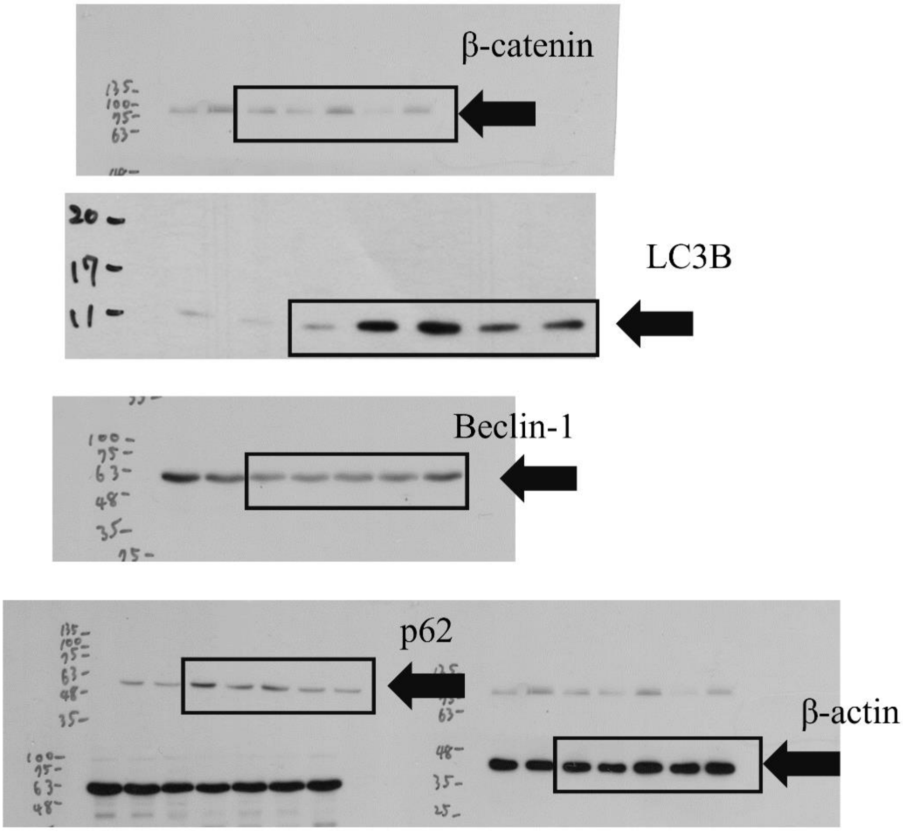

Figure 5. (A)

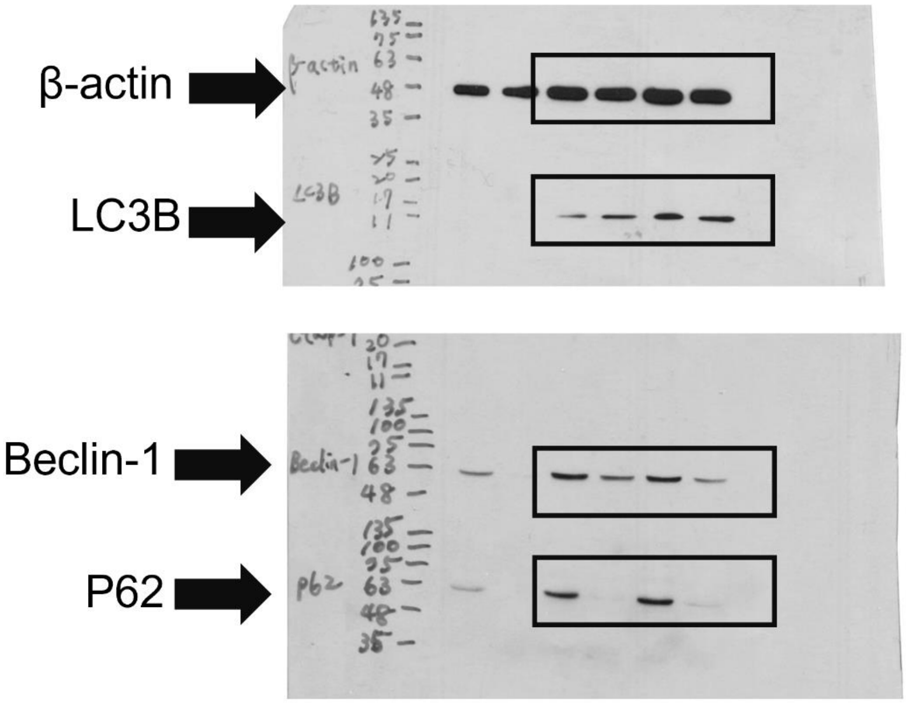

Figure 5. (B)

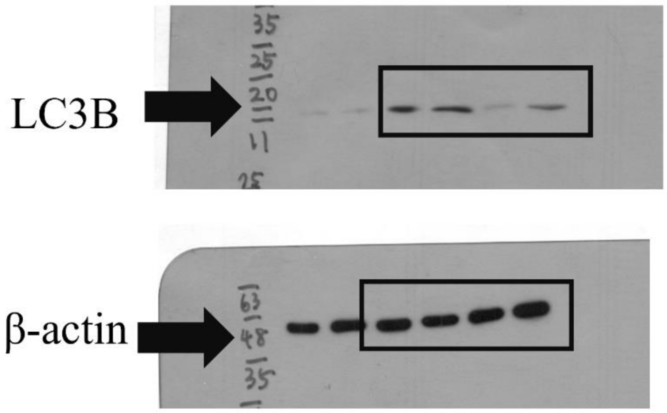

Supplement: S1 Raw images — (ZIP) [file pone.0272797.s003.zip › S1_Raw image.pdf]
